# A randomised trial of Mindfulness-based Social Work and Self-Care with social workers

**DOI:** 10.1007/s12144-023-04410-w

**Published:** 2023-02-25

**Authors:** Alan Maddock, Karen McGuigan, Pearse McCusker

**Affiliations:** 1 School of Social Policy, Social Work and Social Justice, University College Dublin, Dublin 4, Ireland; 2 School of Nursing and Midwifery, Queen’s University Belfast, Belfast, Northern Ireland; 3 School of Social and Political Science, The University of Edinburgh, Edinburgh, Scotland

**Keywords:** Social Work, Self-Care, Stress, Burnout, Mental Health

## Abstract

**Abstract:**

The primary objective of this study was to examine the effects of a bespoke and innovative six-week online Mindfulness-based Social Work and Self-Care (MBSWSC) programme on the stress, feelings of burnout, anxiety, depression, and well-being of a sample of social workers. This secondary objective was to examine the effectiveness of MBSWSC at improving a number of potentially important mindfulness-based programme mechanisms of action, including mindfulness, attention regulation (decentering), acceptance, self-compassion, non-attachment, aversion, worry and rumination. A randomised controlled trial with repeated measures (pre-post intervention) was conducted to evaluate the effects of MBSWSC against an active control. The active control was a modified mindfulness-based programme which focussed on supporting increases in mindfulness and self-compassion in social workers with a view to improving the same primary study outcomes. Sixty-two participants were randomly allocated to MBSWSC (n = 33) or the active control (n = 29). When compared to the active control group, the MBSWSC programme was found to be significantly superior at improving stress, emotional exhaustion, anxiety, and depression. MBSWSC was also superior to the active control at improving acceptance, mindfulness, non-attachment, attention regulation (decentering) and worry of the social workers in this study. The results suggest that MBSWSC is a very useful therapeutic programme, which has the capacity to improve a range of important mental health and well-being outcomes for social workers. The results also indicate that the MBSWSC programme has the capacity to improve a range of important mindfulness-based mechanisms of action.

**Trial registration:**

URL: https://www.clinicaltrials.gov; Unique identifier: NCT05519267 (retrospectively registered).

## Introduction

Social worker health and well-being has been a focus for research with the impact of working conditions and work-related stress and burnout of particular interest (Beer et al., 2020). Social work is a stressful occupation with many aspects of the job proving stressful or, at times, overwhelming for practitioners dealing with substantial caseloads, bureaucratic structures, reducing resources, constant policy changes, staff shortages, client trauma, public scrutiny, and a blame culture (Kinman et al., 2020; Turley et al., 2021). Perhaps it is unsurprising that social workers have been found to be at higher risk of stress and ultimately burnout because of their job role, particularly when compared to other occupations (Kinman et al., 2020). Indeed, Turley et al (2021) point to evidence suggesting UK social workers have an average working life span of < 8 years compared to their healthcare counterparts in nursing (16 years) and medicine (25 years). The current COVID pandemic has served to intensity pressures, adversely impacting the well-being of frontline/essential health and social care practitioners (Hosseinzadeh Asl, 2021). It is worrying that negative physical and psychological outcomes for social workers may be recognised and accepted as ‘*consequences of involvement in the social work profession*’ (Crowder & Sears, 2017, p17). For example, stress is not only an indicator of burnout, but can also impact levels of depression, in turn impacting overall functioning and physical health (Belvederi Murri et al., 2019). Despite the awareness of the impact of the social work role, work-related stress, and burnout, it remains concerning that there is an apparent lack of evidence on the implementation and effectiveness of interventions to address or mitigate these concerns and improve outcomes for social workers (Beer et al., 2020). The literature reflects that the fact the prevalence and severity of negative effects among social workers persists suggests that current ‘*prevention and intervention strategies are ineffective’* (Beer et al., 2021, p317). A systemic approach, embedded in social work policy and practice, to support and improve social worker well-being is needed (Kinman et al., 2020).

Mindfulness has emerged as a potential approach which appears to have promise in improving recovery from and adaptation to stress, improving cognitive and emotional flexibility and behavioural responses to stress whilst serving to promote resilience (Craigie et al., 2016). Mindfulness has been shown to have positive outcomes for those working in health and social care; promoting well-being, increasing self-care and self-compassion, reducing psychological distress, lowering stress, and preventing burnout (Maddock et al., 2021; Vonderlin et al., 2020). Self-care is recognised as a key component for social worker well-being with the National Association of Social Workers endorsing the adoption of self-care training and techniques to counteract associated negative health effects (NASW, 2020). The literature supports the development and use of self-care among social workers due to the associated benefits for social workers, their clients, their organisations, and the profession (Hosseinzadeh Asl, 2021). Mindfulness, developed from Buddhist traditions (Beer et al., 2020; Lynn & Mensinga, 2015), is commonly defined as ‘*paying attention…on purpose, in the present moment and non-judgementally’* (Kabat-Zinn, 1994, p.4). This mindfulness of the ‘present’ has been found to benefit social workers and their practice through improved attention, better self-awareness, greater empathy and compassion, increased calmness and supporting the development of alternative strategies for better daily functioning and well-being (Lynn & Mensinga, 2015).

Currently, two main types of mindfulness-based programmes (MBPs) dominate mindfulness provision: Mindfulness-based Stress Reduction (MBSR: Kabat-Zinn, 1990) and a derivative of this, Mindfulness-based Cognitive Therapy (MBCT: Segal et al, 2002). Use of MBPs in patient and professional populations have evidenced positive outcomes including improved physical and psychological well-being, reduced stress, and burnout (Goodman & Schorling, 2012; Maddock & Blair, 2021; Maddock et al., 2019). However, to date, many of the mindfulness intervention studies have focused on healthcare or student populations (Calcagni et al., 2021; Maddock et al., 2021). Although the potential benefits of mindfulness have been touted for social workers, particularly in recent times, there remains a lack of evidence due to few intervention studies targeting social workers (Kinman et al., 2020). There is a scarcity of studies that are focused on social workers, social work practice, the unique stressors it presents and how social workers cope with this stress; with a need for high quality randomised controlled trials to inform future provision and add to the evidence base in this area (Beer et al., 2020; Crowder & Sears, 2017).

Due to the dominance of MBSR and MBCT programmes, most MBPs are generally delivered in an 8-week format, as this is considered to allow appropriate time for participants to understand, develop, refine, and practice mindfulness (Isbel et al., 2020). Existing programmes such as MBSR and MBCT require significant time commitments from participants which, it is argued, may not be suitable for professionals in practice (Craigie et al., 2016; Isbel et al., 2020). This reflects current calls for tailored MBPs for differing circumstances or occupations which draw on learning from existing standardised programmes but see these protocols refined and adapted to reflect specific occupational needs and organisational characteristics (Calcagni et al., 2021). The evidence available on the effectiveness of briefer MBPs is still scant (Hosseinzadeh Asl, 2021); however there is some evidence to support the use of briefer mindfulness programmes to overcome existing barriers to effectiveness in health and social care whilst offering similar benefits as those seen in the more traditional 8-week programmes (Bartlett et al., 2017; Thomas, 2017). The use of non-traditional delivery methods e.g., online delivery, also seem to be more important among this population (Xu et al., 2021). Given the unique demands and pressures which social work presents, the above should be key considerations for MBPs targeting social workers. In response to the need for a more accessible but targeted MBP for social workers, the bespoke and innovative six-week online Mindfulness-based Social Work and Self-Care (MBSWSC) programme was developed (Maddock et al., 2021). MBSWSC differs from other MBPs by being underpinned by the clinically modified Buddhist psychological model (CBPM), which is an evidence-informed theory of how MBPs, which include increased psychoeducation, could help to improve the feelings of stress, burnout, anxiety, depression, and well-being deficits that can result from social work practice (Maddock, 2022). This theory differs from other models of mindfulness, by focussing on the effects that six mindfulness mechanisms of action (CBPM domains); mindfulness, acceptance, attention regulation (decentering), self-compassion, non-attachment, and non-aversion, could have on these important mental health and well-being outcomes for social workers (Maddock, 2022). The development of each of these six domains (which are developed in MBSWSC through mindfulness, psychoeducation and reflective practice activities) can operate individually, or collectively, to support social workers to move away from the use maladaptive avoidant stress coping strategies (e.g., denial), and to instead use more adaptive approach-oriented stress coping stress strategies (e.g., reflecting on, and engaging with the source of stress, and cognitive, emotional and physical sequalae that accompanies it) (Maddock, 2022). The CBPM outlines how increases in each of these domains, and in tendencies towards the use of approach-oriented stress coping strategies, leads to social workers experiencing reduced negative thinking (e.g., worry and rumination) and improvements in stress, burnout, anxiety, depression, and well-being (Maddock, 2022).

We will examine the effects of the online MBSWSC programme among a sample of social workers in Northern Ireland. This MBSWSC programme has already seen positive effects among social work students, improving attention regulation (decentering), mindfulness, acceptance, self-compassion, non-attachment, and aversion (experiential avoidance) among social work students; as well as increasing non-judgement, empathy, and observation in social work practice (Maddock et al., 2021). Positive effects of MBSWSC on student stress, emotional exhaustion, personal achievement, anxiety and well-being were also evidenced as a result (Maddock et al., 2021).

The effectiveness of the MBSWSC programme will be assessed against an active control, which was a more general MBP which focussed on the development of mindfulness and self-compassion. This study will adopt a randomised controlled trial methodology to examine:

- The effectiveness of the MBSWSC programme at improving social worker stress, feelings of burnout, anxiety, depression, and well-being.
- Effectiveness of MBSWSC at improving mindfulness, attention regulation (decentering), acceptance, self-compassion, non-attachment, aversion, worry and rumination.
- Differences between MBSWSC and a more general MBP.

It is hypothesised that participants in the MBSWSC programme will report improvements in the assessed primary and secondary outcomes. It is also hypothesised that MBSWSC participants will report greater, significant, changes in assessed outcomes when compared to participants on the active control MBP.

## Methods

### Design and setting

A randomised controlled trial with repeated measures (pre-post intervention) was conducted to evaluate the effects of a six-week, online MBSWSC programme against an active control. We utilized an active control condition to control for non-specific variables such as receiving attention, being part of a treatment programme, or group-related factors. We also controlled the amount of home mindfulness practice given to both groups, with the MBSWSC and the active control group receiving a 3-min breathing exercise and 20-min body scans of the same duration over a six-week period. This was due to other reported studies e.g., Greenberg et al. (2018) finding that higher amounts of self-reported practice being associated with positive outcomes. A list of computer-generated random numbers was utilised to assign participants to experimental and control groups. Group allocation was carried out by the study researcher (KMG).

### Ethical approval

Ethical approval for the study was granted by the Research Ethics Committee, School of Social Sciences, Education and Social Work at Queen’s University Belfast (REF_204_2021). All social workers who expressed an interest in the study received written information about the study, with the study researcher available to deal with any additional questions. Social workers who chose to participate in the study provided written, informed consent prior to randomisation. The trial was registered with the clinicaltrials.gov registry, with a unique identifier assigned: NCT05519267.

### Sample

**Fig. 1:**
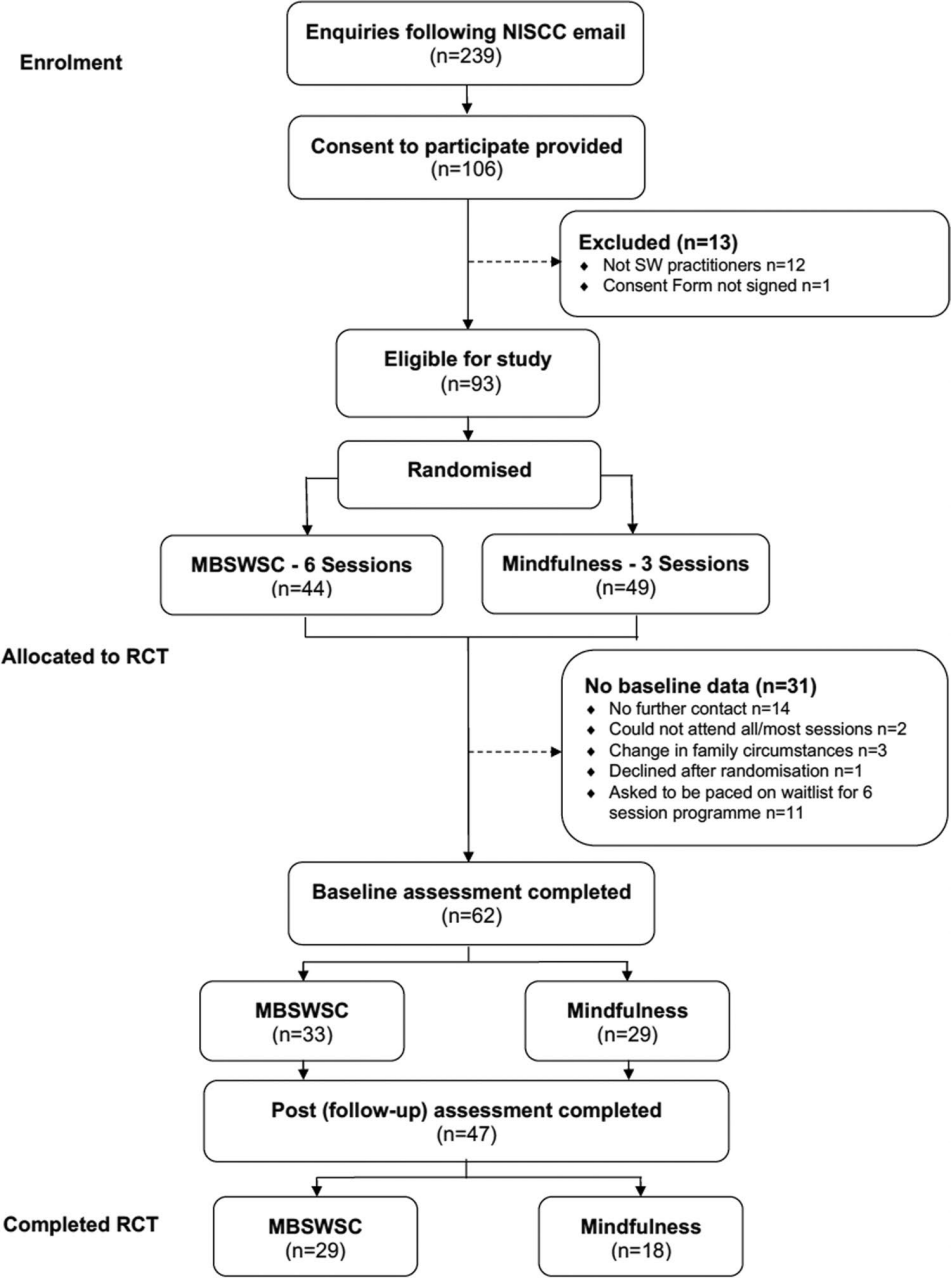
Participant flow: enrolment to completion

The sample comprised of social workers drawn from various agencies and Trusts throughout Northern Ireland. Inclusion criteria were: frontline social work practitioner; working in Northern Ireland; aged 18 years and over. Exclusion criteria were: Working in strategic social work roles with no contact with clients; working outside of Northern Ireland; retired/no longer working in social work. Figure 1 illustrates the study participant flow from recruitment to completion.

### Procedure

Recruitment took place across September–October 2021. Recruitment of social workers for this study was facilitated by Northern Ireland Social Care Council (NISCC), the regulator for standards in social work and social care in Northern Ireland. NISCC maintain a register for practicing social workers in NI and contact their members regularly to highlight appropriate training opportunities or initiatives which may aid their professional practice. NISCC contacted all social workers included on their registered database to inform them about the mindfulness programmes. Those who were interested in finding out more about the study contacted the study researcher who supplied them with participant information sheet and appropriate consent forms. After obtaining written informed consent, participants who met the inclusion criteria were randomised to either the experimental or control group.

### Experimental group (Mindfulness-based Social work and Self-care)

Those assigned to the experimental group received the six-week MBSWSC programme. MBSWSC is a unique mindfulness-based programme for social work and self-care which has been specifically developed for online delivery. The MBSWSC programme is embedded within key cognitive and emotion regulation, and stress coping theory, the CBPM (Maddock, 2022); and guided by an associated MBSWSC protocol. Each session was facilitated by two trained mindfulness practitioners, who are also qualified social workers and thus had insight into the challenges faced by colleagues participating in the programme. The programme comprised of six sessions: 1. Introduction to mindfulness and the CBPM theory underpinning the MBSWSC programme; 2. Stress, the thinking process, avoidant coping, and decentering; 3. Attachment, aversion, negative thinking, and approach coping; 4. Acceptance, self-compassion, the thinking process, and approach coping; 5. Mindfulness as a support to anti-oppressive social work practice; 6. Programme review and embedding mindfulness in everyday life and social work practice. The MBSWSC programme was delivered online via Microsoft Teams on a weekly basis, with weekly sessions lasting approximately 1.5 h. The programme utilised a number of bespoke body scan meditations, which participants then practiced at home : 1) a general body scan meditation, 2) a self-compassion body scan meditation (including the writing of a self-compassionate letter between weeks 4 and 5), 3) an acceptance body scan meditation, 4) an aversion and non-attachment body scan, and 5) a attention regulation (decentering) body scan to help participants develop, refine and practice mindfulness skills/techniques. These homework activities took approximately 20–30 min to complete, 6 days per week over a 6-week period.

### Active control group (Mindfulness and Self-compassion)

The key components of this three-session Mindfulness and Self-Compassion (MSC) programme originated from a structured 8-week Mindfulness Based Living Course (MBLC: Choden & Regan-Addis, 2018) which teaches self-compassion as a discrete element distinct from, but related to, the practice of mindfulness. The main aim of the MSC programme was to provide a condensed MBP, embedded in theory; introducing participants to the concepts and practices of mindfulness with a focus on self-compassion. The structure of the MSC programme mirrors that of the longer MBLC: comprising an outline of a basic mental model and concepts, experience of practice, and personal reflection. Three key practices were chosen for their relevance and utility to social workers: 1. Body scan to enable a shift of attention from mind to body, and to provide a basis for the development of embodied reflexivity useful to the practice of social work, 2. a 3-Minute Breathing (3MBS) Space, and 3 a Self-Compassion Break (SCB). In addition, visual mnemonics for the 3MBS and SCB were provided in the hope that participants will learn and use these helpful practices in their day-to-day lives. The MSC programme was also delivered over a 6-week period. The programme was delivered online, via MS Teams on a fortnightly basis, with sessions lasting for approximately 1 h. The programme also utilised brief homework activities in the form of: 1) a general body scan meditation, 2) 3-min breathing spaces and, 3) self-compassion breaks, to help participants develop, refine and practice mindfulness skills/techniques. These activities also took approximately 20–30 min to complete, 6 days per week over a 6-week period.

### Measures

Self-report measures were administered pre-and-post intervention to assess programme efficacy via changes between, and within, the experimental and control group. Demographic information was gathered, at baseline only, on participants’ age, sex, and job role. Scale reliabilities were calculated using responses provided at baseline.

#### *The Perceived Stress Scale (PSS:*Cohen et al., 1983*):*

The PSS is a widely used measure to assess the extent to which individuals perceive situations in their lives to be stressful; and was originally developed to assess stress within community samples/cohorts (Cohen et al., 1983). The PSS has been shown to be a valid and reliable measure of perceived stress in occupational groups e.g., a study conducted among university teachers highlighted adequate concurrent validity and good reliability (0.86) (Siqueira et al., 2010); with a study conducted among social work students also confirming high internal consistency (α = 0.91) (Maddock et al., 2021). The measure comprises 10 items, scored on a five-point Likert scale (0 = never;4 = very often). Higher scores are indicative of higher levels of stress. Among study participants, the reliability of the scores on the PSS was found to be acceptable (Cronbach’s α = 0.88).

#### *The Maslach Burnout Inventory (MBI:* Maslach et al., 1996*):*

The MBI is a reliable and valid measure of work-related burnout utilised in various occupational samples, including social workers (Crowder & Sears, 2017). Convergent and discriminant validity of MBI has been confirmed in a range of populations (Maslach et al., 1996, 2001). The most widely used burnout inventory, the MBI comprises 22 items scored on a 7-point Likert scale (0-never; 6 = everyday). The MBI has 3 subscales: emotional exhaustion, depersonalisation/loss of empathy, and personal achievement. Maslach et al. (2001) confirm moderate to high reliability on the MBI subscales reporting Cronbach’s alpha of 0.90, 0.79 and 0.71 for emotional exhaustion, depersonalisation, and personal accomplishment respectively. A recent study conducted among social work students reported an α of 0.81, 0.71 and 0.85 for emotional exhaustion, depersonalisation, and personal accomplishment respectively. Scores of ≤ 17 on the emotional exhaustion scale are indicative of low-level emotional exhaustion; whilst scores of 18–29 suggest moderate level emotional exhaustion and scores ≥ 30 indicate high-level emotional exhaustion. On the depersonalisation/loss of empathy subscale, low-level depersonalisation is indicated by a score of ≤ 5; whilst scores of 6–11 suggest moderate depersonalisation and scores ≥ 12 indicate high-level depersonalisation. Lastly, on the personal achievement subscale, low-level personal achievement is indicated by a score of ≥ 40; whilst scores of 34–39 suggest moderate level personal achievement and scores ≤ 33 indicate high-level personal achievement. The Cronbach’s alpha for this study was 0.91, 0.69 and 0.75 for emotional exhaustion, depersonalisation, and personal accomplishment respectively.

#### *The Hospital Anxiety and Depression Scale (HADS:* Zigmond & Snaith, 1983*):*

The HADS is a reliable and valid measure which has been used to assess anxiety and depression among out-patients and in occupational settings (Sanne et al., 2003). A review of studies reporting on the validity of HADS highlighted the good to very good convergent validity of the scale; whilst reliability was reported to range from 0.68 to 0.93 across included studies (Bjelland et al., 2002). The HADS comprises 14 items assessing severity of anxiety (HADS-A) and depression (HADS-D), with 7 items in each subscale. It is scored on a 4-point Likert scale (0–3) with anxiety and depression subscale scores ranging from 0 to 21. Subscale scores of 0–7 are considered normal, scores of between 8-10 indicate the presence of a mild disorder, whilst scores of 11 or more are classified as moderate to severe. The Cronbach’s alpha for this study was 0.80, 0.78 and 0.73 for HADS, HADS-A and HADS-D respectively.

#### *Warwick-Edinburgh Mental Well-being Scale (WEMWBS: *Tennant et al, 2007*):*

The WEMWBS is a reliable and valid measure of mental well-being (Tennant et al., 2007). In their work to develop and validate the WEMWBS, Tennant et al. (2007) confirmed the scale displayed good content validity and a high test–retest reliability of 0.83. The scale comprises 14 items, scored on a 5 point-Likert scale; with lower scores indicative of poorer mental well-being. The Cronbach’s alpha for this study was 0.86.

#### *The Southampton Mindfulness Questionnaire (SMQ: *Chadwick et al., 2008*):*

The SMQ is a 16-item measure assessing elements of mindfulness in response to unpleasant thoughts and images (Chadwick et al., 2008). The SMQ has been found to have adequate concurrent and discriminant validity (Chadwick et al., 2008). The SMQ is scored on a 7-point Likert scale (0 = Disagree totally; 6 = Agree totally). High scores on the SMQ are indicative of higher levels of mindfulness, with scores ranging from 0 to 96. A recent study conducted among social work students reported high internal consistency (α = 0.94) (Maddock et al., 2021). Among study participants, the reliability of the scores on the SMQ was found to be acceptable (Cronbach’s α = 0.87). This scale also delves into the components of mindfulness comprising 4 subscales measuring Mindful Observation (SMQ-MO); Letting Go/Non-attachment (SMQ-LG); Aversion (SMQ-Av); and Non-judgement (SMQ-NJ). The Cronbach’s alpha for these subscales, in this study, were 0.75; 0.76; 0.82; 0.79 respectively.

#### *The Experiences Questionnaire – Decentering (EQ-D: *Fresco et al., 2007*):*

The EQ-D is a measure of decentering, reflecting an individual’s ‘*capacity to take a detached view of one’s thoughts and emotions’* (Fresco et al., 2007, p.234). Convergent and discriminant validity of the measure was confirmed in a series of studies by Fresco et al. (2007). A further study confirmed good construct validity, as well as high reliability (α = 0.81) of the scale (Gregório et al., 2015). This 11-item measure is scored on a 5-point Likert scale (1 = never; 5 = always), with scores ranging from 11–55. Higher scores on this measure are indicative of higher levels of attention regulation (decentering). The Cronbach’s alpha for this study was 0.84.

#### *The Philadelphia Mindfulness – Acceptance Subscale (PHLMS-A:* Cardaciotto et al., 2008*):*

The PHLMS-A is a 10-item reliable and valid measure of the key mindfulness trait of acceptance (Cardaciotto et al., 2008). Cardaciotto et al. (2008) confirmed the convergent and discriminant validity of the *PHLMS-A*; whilst Maddock et al. (2021) reported good scale reliability among a sample of social work students, with a Cronbach’s alpha of 0.87. The scale is scored on a 5-point Likert scale (1 = never; 5 = very often); with total scores ranging from 10–50. Lower scores on the PHLMS-A are indicative of greater levels of acceptance. The Cronbach’s alpha for this study was 0.89.

#### *The Self-Compassion Scale (SCS: *Neff, 2003*):*

The SCS is a reliable and valid measure of self-compassion (Neff, 2003). Neff (2003) confirmed good construct validity and scale test–retest reliability (α = 0.93) of the scale. It is a 26-item measure, scored on a 5-point Likert scale (1 = almost never; 5 = almost always). Higher scores are indicative of higher levels of self-compassion. Among study participants, the reliability of the scores on the SCS was found to be acceptable (Cronbach’s α = 0.87).

#### *Penn State Worry Questionnaire (PSWQ; *Meyer et al., 1990*):*

The PSWQ is a 16-item, reliable and valid measure of worry; more specifically the pervasiveness, intensity, and uncontrollability of worry (Startup & Erickson, 2006). The scale displays good discriminant validity and internal consistency (Brown et al., 1992). The scale is scored on a 5-point Likert scale (1 = not at all typical of me; 5 = very typical of me). Scores on the PSWQ range from 16–80, with higher scores indicative of a higher levels of pathological worry (Startup & Erickson, 2006). Among study participants, the reliability of the scores on the PSWQ was found to be acceptable (Cronbach’s α = 0.95).

#### *Rumination Reflection Questionnaire (RRQ: *Trapnell & Campbell, 1999*):*

Rumination, the extent to which individuals tend to participate in repetitive or recurring thoughts about their past experiences, was measured using the Rumination subscale from the RRQ. Trapnell and Campbell (1999) reported the rumination subscale exhibited good convergent and discriminant validity. Whilst Maddock et al. (2021) reported the subscale has moderate reliability among a sample of social work students. This 12-item Rumination subscale was scored on a 5-point Likert scale (1 = strongly disagree; 5 = strongly agree), with scores ranging from 12–60. Higher scores on the scale are indicative of higher levels of/engagement in rumination. The Cronbach’s alpha for this study was 0.94.

## Results

Figure 1 illustrates the study participant flow from recruitment to completion. Of the 239 enquiries/expressions of interest received, 106 participants provided informed consent to participate in the study. Of these, 93 were eligible for inclusion in the study. 44 were allocated to the experimental group (MBSWSC) and 49 to the active control group (MSC). 62 participants completed baseline assessment measures, with 47 completing post-intervention assessments. There were no complaints, difficulties, ill/unintended effects reported by study participants. The study comprised 55 females (88.7%) and 7 males (12.3%). Ages in the sample ranged from 24 to 64 years (M = 44.44; SD = 10.01). Table 1 illustrates this by group.

**Table 1:** Demographics

	MBSWSC (n = 33)	MSC (Control) (n = 29)
Age, years M(SD)[min–max]	43.3(9.16) [24–59]	45.7(10.9) [26–64]
Female *n* (%)	31 (93)	24 (83)
Male* n* (%)	2 (7)	5 (17)

### MBSWSC and MSC (Between groups)

Data were specified and tested in SPSS using PROCESS (Hayes, 2013), with analysis of covariance (ANCOVA) used to explore differences between groups at follow-up (post-intervention) whilst controlling for any baseline differences. Within the specified ANCOVA analyses, planned contrasts and bootstrapping were also specified to further explore between group differences and ensure more robust reporting (Field, 2013). Significant differences were found between the MBSWSC and MSC groups across 10 of the 17 measures assessed (See Table 2). The MBSWSC group reported a large significant reduction in stress scores compared to the MSC group when baseline scores were controlled for at follow-up *F*(1,56) = 9.876, *p* = 0.003,η2 = 0.15. Planned contrasts revealed that those in the MBSWSC had significantly reduced stress scores compared to those in the MSC group, *t*(56) = -3.141, *p* = 0.005. MBSWSC group also reported a large significant reduction in emotional exhaustion: *F*(1,53) = 8.756, *p* = 0.005,η2 = 0.142, depression: *F*(1,55) = 9.931, *p* = 0.003,η2 = 0.153, and worry: *F*(1,57) = 12.00, *p* = 0.001,η2 = 0.174 scores compared to MSC group when baseline scores were controlled for at follow-up. Planned contrasts revealed that those in the MBSWSC had significantly reduced emotional exhaustion: *t*(53) = -2.959, *p* = 0.005; depression: *t*(55) = -3.151, *p* = 0.003; and worry: *t*(57) = -3.464, *p* = 0.001 compared to those in the MSC group.

**Table 2:** Means, standard deviations (in parentheses), and ANCOVA test statistics

	MBSWSC M(SD)	MSC (Control) M(SD)	*F*	*p*	η2
Stress (PSS)					
Pre-intervention	20.72 (5.90)	18.56 (6.62)			
Post-intervention	14.34 (4.42)	16.85 (6.55)	9.88	0.00	0.15
Burnout – Emotional exhaustion (MBI-EE)					
Pre-intervention	19.90 (9.67)	18.46 (9.57)			
Post-intervention	13.60 (6.90)	17.19 (9.03)	8.76	0.01	0.14
Burnout – Depersonalisation (MBI-D)					
Pre-intervention	10.77 (6.65)	10.42 (7.03)			
Post-intervention	8.42 (7.11)	10.38 (8.30)	2.99	0.09	0.05
Burnout – Personal Achievement (MBI-PA)					
Pre-intervention	33.90 (7.43)	31.60 (7.69)			
Post-intervention	36.43 (5.35)	32.80 (8.54)	2.34	0.13	0.04
Anxiety (HADS-A)					
Pre-intervention	9.38 (3.67)	8.26 (3.19)			
Post-intervention	6.66 (2.95)	7.67 (3.23)	7.23	0.01	0.11
Depression (HADS-D)					
Pre-intervention	5.25 (2.90)	4.73 (2.47)			
Post-intervention	2.94 (2.49)	4.04 (2.58)	9.93	0.00	0.15
Well-being (WEMWBS)					
Pre-intervention	46.64 (5.25)	46.71 (5.73)			
Post-intervention	51.03 (6.85)	49.42 (7.21)	1.14	0.29	0.02
Mindfulness (SMQ)					
Pre-intervention	45.64 (14.06)	47.76 (12.85)			
Post-intervention	58.48 (13.55)	51.31 (18.55)	8.15	0.01	0.12
Decentering/Attention Regulation (EQ- D)					
Pre-intervention	33.03 (4.34)	33.62 (4.75)			
Post-intervention	38.81 (5.13)	36.31 (7.17)	5.12	0.03	0.08
Acceptance (PHLMS-A)					
Pre-intervention	33.33 (6.51)	30.41 (6.46)			
Post-intervention	29.58 (6.54	31.41 (7.63)	4.10	0.05	0.07
Self-Compassion (SCS)					
Pre-intervention	34.47 (9.12)	32.04 (7.68)			
Post-intervention	39.72 (7.18)	35.43 (8.60)	3.75	0.06	0.06
Mindful Observation (SMQ-MO)					
Pre-intervention	12.24 (4.49)	13.14 (3.53)			
Post-intervention	15.45 (3.55)	14.59 (4.35)	2.01	0.16	0.03
Letting Go/Non-Attachment (SMQ- LG)					
Pre-intervention	10.03 (4.10)	10.35 (4.58)			
Post-intervention	13.91 (4.05)	11.79 (5.37)	4.87	0.03	0.08
Aversion (SMQ-Av)					
Pre-intervention	12.52 (3.60)	12.90 (3.66)			
Post-intervention	14.97 (3.73)	13.83 (5.48)	2.57	0.11	0.04
Non-Judgement (SMQ-NJ)					
Pre-intervention	11.06 (4.31)	11.97 (3.93)			
Post-intervention	14.36 (3.78)	13.24 (5.40)	5.36	0.02	0.08
Worry (PSWQ)					
Pre-intervention	56.69 (13.02)	53.07 (12.77)			
Post-intervention	46.38 (11.04)	49.71 (13.56)	12.00	0.00	0.17
Rumination (RRQ)					
Pre-intervention	42.12 (7.65)	44.00 (8.19)			
Post-intervention	35.85 (7.62)	40.10 (10.13)	2.61	0.11	0.04

The MBSWSC group reported moderate or medium reductions in anxiety compared to the MSC group when baseline scores were controlled for at follow-up, *F*(1,56) = 7.228, *p* = 0.009,η2 = 0.114. Planned contrasts revealed that those in the MBSWSC had significantly reduced anxiety scores compared to those in the MSC group, *t*(56) = -2.688, *p* = 0.009. MBSWSC group also reported a medium significant improvement in mindfulness: *F*(1,59) = 8.147, *p* = 0.006,η2 = 0.121; attention regulation (decentering): *F*(1,58) = 5.123, *p* = 0.027,η2 = 0.081; acceptance: *F*(1,59) = 4.100, *p* = 0.047,η2 = 0.065; non-attachment: *F*(1,59) = 4.872, *p* = 0.031,η2 = 0.076; and non-judgement: *F*(1,59) = 5.358, *p* = 0.024,η2 = 0.083 when baseline scores were controlled for at follow-up. Planned contrasts revealed that those in the MBSWSC had significantly improved mindfulness: *t*(59) = 2.854, *p* = 0.006; attention regulation (decentering) *t*(58) = 2.263, *p* = 0.027; acceptance: *t*(59) = 2.025, *p* = 0.047; non-attachment: *t*(59) = 2.207, *p* = 0.031; and non-judgement: *t*(59) = 2.315, *p* = 0.024 compared to those in the MSC group. No other significant between groups differences were found. However, to better understand the impact of both interventions, within group differences were explored to examine changes between pre-and-post measures in both groups.

### MBSWSC and MSC (Within groups)

Differences were also found within groups, with paired-samples t-tests evidencing significant changes across pre-post assessment in both the MBSWSC and MSC groups. On average MBSWSC participants had lower levels of stress post-intervention (M = 14.343; *SE* = 0.787) than pre-intervention (M = 20.718; *SE* = 1.175). This difference (-6.375, BCa 95% CI [-8.366;-4.383] was statistically significant *t*(31) = -6.530, *p* = 0.000. MSC participants also reported lower levels of stress post-intervention. This difference: -1.703, BCa 95% CI [-3.568;0.161] was not statistically significant *t*(26) = -1.878, *p* = 0.072. MBSWSC participants reported lower levels of emotional exhaustion post-intervention. This difference: -6.300, BCa 95% CI [-8.810;-3.790] was statistically significant *t*(29) = -5.134, *p* = 0.000. Although a reduction in emotional exhaustion was found post intervention within the MSC group, this was non-significant, *t*(25) = -0.945, *p* = 0.354. On the burnout depersonalisation subscale, MBSWSC participants reported lower levels post-intervention. This difference: -2.355 BCa 95% CI [-4.292;-0.06] was statistically significant *t*(28) = -2.11, *p* = 0.04. Although a reduction in depersonalisation was found post intervention within the MSC group, this was non-significant, *t*(23) = -0.038, *p* = 0.966. A similar pattern was found with anxiety and depression. MBSWSC participants reported lower levels of anxiety post-intervention. This difference: -2.718, BCa 95% CI [-3.867;-1.570] was statistically significant *t*(31) = -4.829, *p* = 0.000. Although a reduction in anxiety was found post intervention within the MSC group, this was non-significant, *t*(26) = -1.507, *p* = 0.144. MBSWSC participants reported significantly lower levels of depression post-intervention *t*(31) = -6.319, *p* = 0.000. A non-significant reduction in depression was evidenced in MSC, *t*(26) = -2.031, *p* = 0.053. With regards to well-being, MBSWSC participants had higher levels of well-being post-intervention (M = 51.030; *SE* = 1.193) than pre-intervention (M = 46.636; *SE* = 0.914). This difference (4.394, BCa 95% CI [2.088;6.700] was statistically significant *t*(32) = 3.881, *p* = 0.000. MSC participants also reported higher levels of well-being post-intervention. This difference: 2.714, BCa 95% CI [0.432;4.997] was statistically significant *t*(27) = 2.440, *p* = 0.022. On average MBSWSC participants had higher levels of mindfulness post-intervention (M = 58.485; *SE* = 2.358) than pre-intervention (M = 45.636; *SE* = 2.447). This difference (12.848, BCa 95% CI [8.829;16.868] was statistically significant *t*(32) = 6.511, *p* = 0.000. MSC participants also reported higher levels of mindfulness post-intervention. This difference: 3.552, BCa 95% CI [-1.568;8.672] was not statistically significant *t*(28) = 1.421, *p* = 0.166. MBSWSC participants reported higher levels of attention regulation (decentering) post-intervention. This difference: 5.781, BCa 95% CI [4.164;7.398] was statistically significant *t*(31) = 7.292, *p* = 0.000. Improvements in attention regulation (decentering) were also found post intervention within the MSC group, this was statistically significant, *t*(28) = 2.500, *p* = 0.019. MBSWSC participants reported significantly improved levels of acceptance post-intervention *t*(32) = 3.758, *p* = 0.001. A non-significant improvement in acceptance was evidenced in MSC, *t*(28) = 1.224, *p* = 0.231. MBSWSC participants reported significantly higher levels of self-compassion post-intervention *t*(31) = 5.740, *p* = 0.000. A significant improvement in self-compassion was also evidenced in MSC, *t*(27) = 3.078, *p* = 0.005. Significant improvements post-intervention, were also noted in non-attachment (*t*(32) = 6.420, *p* = 0.000); aversion (*t*(32) = 3.657, *p* = 0.00); mindful observation (*t*(32) = 4.923, *p* = 0.000); non-judgement (*t*(32) = 6.187, *p* = 0.000) among the MBSWSC group. However, although improvements were evidenced for these factors among the MSC group, these were not statistically significant: non-attachment (*t*(28) = 1.447, *p* = 0.159); aversion (*t*(28) = 1.317, *p* = 0.199); mindful observation (*t*(28) = 1.721, *p* = 0.096); non-judgement (*t*(28) = 2.046, *p* = 0.050). Lastly, significant post-intervention reductions in worry and rumination were noted in MBSWSC group: (*t*(31) = 4.285, *p* = 0.001) and (*t*(31) = -3.617, *p* = 0.000) respectively. Similar significant reductions were found in the MSC group for worry: (*t*(27) = -1.068, *p* = 0.295) and rumination: (*t*(27) = -2.692, *p* = 0.012). No significant differences were found in either group, pre-post intervention, in relation to personal achievement.

## Discussion

This study examined the effectiveness of the MBSWSC programme at improving social worker stress, feelings of burnout, anxiety, depression, and well-being along with a range of potential universal predicting and mediating mechanisms of how MBSWSC might improve these outcomes, including mindfulness, attention regulation (decentering), acceptance, self-compassion, non-attachment, aversion, worry and rumination. Consistent with the hypotheses, this study indicates that MBSWSC improves each of these potential mechanisms of action and each of these outcomes. When compared to the active control group, which focused on developing mindfulness and self-compassion in social workers, MBSWSC was significantly superior at improving stress, emotional exhaustion, anxiety, and depression. The MBSWSC programme was also found to be significantly superior versus the active control at improving acceptance, mindfulness, attachment, attention regulation (decentering), worry and non-judgement.

MBSWSC was found to improve both stress and anxiety versus the active control (MSC) group. This diverges from Hosseinzadeh Asl (2021) which, in the only other RCT on the effectiveness of a mindfulness-based intervention for social workers, found that a 4-week programme did not improve stress or anxiety versus a waitlist control group. The capacity for MBSWSC to improve stress is supported by Crowder and Sears (2017), who in a study examining the effectiveness of an 8-week MBSR programme in a non-randomised controlled trial with social workers found that MBSR significantly improved perceived stress. The difference between MBSWSC and Hosseinzadeh Asl (2021) may be that MBSWSC is of a longer duration (6 versus 4 weeks) and that the MBSWSC programme was developed based on a promising evidence-informed theory of how MBPs might improve stress, feelings of burnout, anxiety, depression, and well-being, the CBPM (Maddock, 2022). It also may be that the additional psychoeducation, derived from the CBPM theory, of how MBPs might impact the study outcomes; social work role plays (allowing the application of the learning to social work practice) and tailoring of the body scanning (e.g. self-compassion and acceptance body scans) to improving the predictor and mediating variables of the CBPM; may have led to significant improvements in these outcomes versus the active control group. This may also account for why it appears to have been more effective than the MBP in Hosseinzadeh Asl (2021).

The moderate reduction in anxiety and the large reductions in depression and stress due to MBSWSC participation is line with a meta-analysis of 209 studies conducted by Khoury et al. (2013) which have found that programmes of a longer duration, such as MBSR and MBCT have moderate to large effects on anxiety, depression, and stress. Spijkerman et al. (2016) conducted a meta-analysis of 15 RCTs which examined the effectiveness of online mindfulness-based programmes for mental health and found that online MBPs had a small but beneficial effect on anxiety and depression and a moderate beneficial effect on stress. Spijkerman et al. (2016) thus supports this study, with the results from this study indicating that MBSWSC, which was developed to be delivered online, may outperform other MBPs (MBSR, MBCT and ACT) on stress, anxiety, and depression, when they are delivered in an online format. The MBSWSC group improvements in both anxiety and depression scores in this study appear to be particularly strong, with participants experiencing a 2.72 decrease in anxiety on HADS-A at T2 and a 2.32 decrease in depression on HADS-D at T2, both of which are above the 1.5-point decrease required to be deemed an important clinical difference score (Puhan et al., 2008). These changes are also in line with the pilot of the MBSWSC programme with social work students, which found that MBSWSC improved the anxiety of social work students by a similar amount on the HADS-A (2.45 points) (Maddock et al., 2021). However, depression change scores did not improve significantly in this previous research study (Maddock et al, 2021). Taking this prior negative finding into account, the current MBSWSC programme was adjusted to include additional psychoeducation and practice focus on attention regulation (decentering) as it was felt that more space could be offered to this potential mechanism of change, particularly due it being identified both theoretically (Maddock, 2022) and empirically as potential mechanism of change in depression (Bennett et al., 2021). This may account for why MBSWSC was significant at improving depression versus the active control in this study and why the social workers in this study experienced higher depression change scores than the social work students did in the pilot MBSWSC programme (Maddock et al., 2021).

In line with Maddock et al. (2021), the present study found that MBSWSC led to a large significant effect on emotional exhaustion compared to the active control group. This study did not see changes on the burnout-personal achievement subscale, either against the active control or within groups. This diverges from Maddock et al. (2021) and may be due to the different career stage in social work in the samples, as the students in Maddock et al. (2021) had lower levels of personal achievement. However, the social workers in this study, though experiencing high rates of stress, still had higher levels of personal achievement, meaning that there was less room for MBSWSC to influence this domain of burnout. MBSWSC did not significantly change depersonalisation versus the active control in this study, however, looking at the within group effects, the social workers within the MBSWSC group did experience a significant change. This differs from Maddock et al. (2021), where students did not experience a significant change in depersonalisation. This again may reflect where both groups were at in their career trajectory, with the social workers in this study, who are having to deal with the complexity of their role during the Covid-19 pandemic, having higher rates of depersonalisation at baseline, meaning that MBSWSC was not restricted by floor effects on this measure as it may have been with social work students in Maddock et al. (2021).

There were no significant differences between the MBSWSC and the active control in both self-compassion and well-being, however both groups experienced significant within group effects. The degree to which both self-compassion and well-being improved for the social workers in the MBSWSC group was similar to that of the social work students who completed the programme in Maddock et al. (2021). The lack of a significant difference between groups on self-compassion may be due to the focus of both programmes (MBSWSC and MSC) on developing mindfulness and self-compassion in social workers. The fact that MBSWSC improved self-compassion to a greater degree than the active control may be due to MBSWSC’s higher number of sessions over the same six-week period, allowing increased psychoeducation to be attained. The MBSWSC group were also given specific self-compassion-based body scans and an additional task of writing a self-compassionate letter as part of their homework practice, with the active control using a more general body scan and self-compassionate breaks. These findings would indicate, supporting Hosseinzadeh Asl (2021) that although longer MBPs, such as MBSWSC, are likely to lead to greater benefits, mindfulness programmes comprising 3–4 sessions of instruction could potentially improve self-compassion and well-being in social workers.

MBSWSC had a greater effect on the rumination and worry levels of social workers than it did on social work students in Maddock et al. (2021). This may be due to the higher rates of rumination and worry experienced by social workers at baseline in this study, meaning that was more room for MBSWSC to effect these potential mechanisms of change. When compared to the active control group, changes in worry were found to be significantly different but changes in rumination were not. However, both groups experienced within groups changes from pre to post programme, with MBSWSC again outperforming the control group on both potential mediators of change (Maddock, 2022). These results are consistent with those of RCTs which have found MBPs to have significant effects on worry and rumination (van Aalderen et al., 2012; Vøllestad et al., 2011). The engagement in the MBSWSC programme led to the social workers in this study experiencing increased capacities (versus the active control) in a number of the important domains of mindfulness set out in the CBPM theory, which underpins MBSWSC (Maddock, 2022), including acceptance, attention regulation (decentering), mindfulness and non-attachment. One other CBPM domain, aversion, improved within the MBSWSC group, but not significantly, versus the control group. The degree of change experienced in each CBPM domain was similar between this study and the changes experienced by social work students in Maddock et al. (2021). These results are also consistent with a range of RCTs with non-clinical and clinical populations, which found the MBPs have significant effects on acceptance, attention regulation (decentering), and mindfulness (Bieling et al., 2012; Maddock et al., 2019; Vøllestad et al., 2011).

The results from this current study, when set against the wider literature, highlight how social worker engagement with MBPs which have shorter and less frequent practices sessions (i.e. 3 sessions held over 6 weeks for 1 h) might support improved self-compassion and well-being. This study however highlights the suitability and acceptability of the MBSWSC programme to social workers. Braun et al. (2019) sensibly pointed out the need for MBPS, such as MBSR and MBCT, to be shortened, and for any adaptations to be based on a theoretical rationale. This would make it more feasible for health and social care workers to fully engage in and complete MBPs. In order to support social worker engagement and compliance with the MBSWSC programme, it took place online, over 6 weeks, with sessions lasting 1.5 h rather than the 2–2.5 h duration of MBSR or MBCT (Carmody & Baer, 2008). As a comparison, an active control, which was theory informed (Choden & Regan-Addis, 2018) and delivered over three 1-h sessions, with the same homework requirements as MBSWSC (with some adjustments relating to the types of body scans engaged in by participants) over the same 6-week period was delivered. Both programmes only required participants to engage in twenty to thirty minutes of homework practice six out of seven days rather than the typically required forty-five minutes (Carmody & Baer, 2008). Interestingly, even though this MSC was shorter in duration and required less time commitment, it experienced a higher rate of attrition (34%) than the MBSWSC programme (12%). By comparison, Nam and Toneatto (2016) found that RCTs in MBPs have high attrition rates, with a mean of 29%. Although more research is needed on exactly why the rates of attrition were so low in the MBSWSC group, the variety and flexibility of body scans on offer, which focused on a wider array of potential mindfulness mechanisms of action e.g., acceptance, may have made the content richer, and more relevant to social workers, than traditional MBPs. The results indicate that not only did MBSWSC outperform the active control across a range of important stress and mental health outcomes, but in a real-world setting, social workers are also more likely remain compliant with a six session MBP programme over six weeks (of 1.5 h duration) than shorter or longer MBPs.

When assessing the results of this study, its limitations should be considered. This study used self-report measures, which are susceptible to common methods bias. Future research could include other measurement methods e.g., physiological measurements of stress. The selection of valid and reliable outcome measures was directed by key evidence in the mindfulness literature. However, although providing a comprehensive understanding of the effectiveness of the MBSWSC and MSC, the number of variables measured required the use of additional analyses to assess and interpret their impact. Although in the analyses used, more robust techniques and related reporting has been used to mitigate some concern, e.g., the use of percentile bootstrap confidence intervals (Caron, 2019; Hayes, 2023), the possibility of a Type 1 error cannot be ruled out. The study involved frontline social workers in Northern Ireland who had consented to participate in the study. Whilst the participants were randomised to the MBSWSC or MSC groups, no additional information was gathered on this cohort. As such, there were no data on participant characteristics or experience e.g., duration of social work career, requested from participants. This is a limitation of the current study as these personal characteristics or experience level may act as moderators for key outcomes such as burnout. Future studies would benefit from the inclusion of such data. The MBSWSC and MSC interventions were facilitated by two trained mindfulness practitioners, who are also qualified social workers. The facilitators were aware of the condition to which the participants had been assigned as they were responsible for the programme delivery. Efforts were made to reduce any potential bias in the process by also ensuring the facilitators were blinded to any data collection or analyses. However, it must be considered that the awareness of the facilitators of the study condition may have introduced potential bias, which should be addressed in any future studies. Although the study design was appropriate to test the study hypotheses, a final limitation of the study lies in its reliance on an active control group, rather than the use of a no-treatment control group. This study was developed to address an identified need in the social work profession, as such the study sought to provide a supportive intervention rather than no intervention in the control setting. Future studies should endeavour to explore the unique impact of the MBSWSC in relation to social workers who receive no supportive intervention. This study’s results need to be replicated in future studies with social workers. Further, it would be important to examine the longer-term effects of MBSWSC to confirm the sustainability of programme effects across time. It would also be useful to examine the effectiveness of the MBSWSC programmes with social workers who work in different practice settings e.g., child protection and mental health. 

Despite these limitations, this study found that MBSWSC is a feasible therapeutic programme which can help to improve the feelings of stress, burnout, anxiety, depression, and well-being deficits that can accompany social work practice. This study also found that MBSWSC improved several key mechanisms of mindfulness, which could help to support social worker self-care, in line with the theory of mindfulness which underpinned the programme, the CBPM. This study’s findings indicate that if social workers complete an MBSWSC programme, they are likely to experience less stress, burnout, along improved mental health and well-being.

## Data Availability

The datasets generated during and/or analysed during the current study are available from the corresponding author on reasonable request.
